# The prognostic role of systemic inflammatory markers in apparent early-stage ovarian cancer

**DOI:** 10.1007/s10147-022-02272-z

**Published:** 2022-11-22

**Authors:** Nicolò Bizzarri, Marco D’Indinosante, Claudia Marchetti, Riccardo Tudisco, Francesca Turchiano, Giovanni Scambia, Anna Fagotti

**Affiliations:** 1 Dipartimento per la salute della Donna e del Bambino e della Salute Pubblica, UOC Ginecologia Oncologica, Fondazione Policlinico Universitario A. Gemelli, IRCCS, Rome, Italy; 2 Università Cattolica del Sacro Cuore, Rome, Italy

**Keywords:** Ovarian cancer, Systemic inflammation, BRCA status, Prognosis, Survival, Early stage

## Abstract

**Background:**

Few studies analyzed the prognostic role of systemic inflammatory markers in early-stage ovarian cancer. The primary endpoint of the present study was to assess the prognostic impact of baseline inflammatory markers in early-stage ovarian cancer. The secondary endpoints were to compare the disease-free survival (DFS) of inflammatory markers with standard risk factors and to correlate these with BRCA mutational status.

**Methods:**

Retrospective, single-center, observational study. Patients with FIGO-stage I–II and IIIA1 epithelial ovarian cancer undergoing primary surgery between 10/2012 and 12/2019 were included. Inflammatory markers were evaluated on the results of the complete blood count and coagulation tests, performed before ovarian cancer surgery. The Receiver Operating Characteristic curve was used to determine the optimal cut-off value of different baseline inflammatory biomarkers for the DFS analysis.

**Results:**

Three hundred fifty-nine patients were included in the study period. Baseline neutrophil–lymphocyte ratio (NLR) ≥ 3 and systemic immune inflammation index (SII, defined as platelet x neutrophil–lymphocyte ratio) ≥ 1000 were associated with worse 3 year DFS and baseline SII ≥ 1000 was associated with worse 3 year OS. BRCA-mutated patients with SII ≥ 1000 and with NLR ≥ 3 had significantly worse DFS compared to SII < 1000 and with NLR < 3. FIGO stage > I was the only independent risk factor for higher risk of recurrence.

**Conclusion:**

SII ≥ 1000 and NLR ≥ 3 were associated with worse 3 year DFS and SII ≥ 1000 was associated with worse 3 year OS. The subgroups of BRCA-mutated patients with higher inflammation markers (SII ≥ 1000 and NLR ≥ 3) were associated with worse DFS. These findings might be helpful to design personalized treatment and more intensive surveillance.

**Supplementary Information:**

The online version contains supplementary material available at 10.1007/s10147-022-02272-z.

## Introduction

Ovarian cancer represents the most lethal gynecologic cancer, with 295,414 new cases estimated and 184,799 deaths in 2018 worldwide; only about 25% of ovarian cancer patients are diagnosed with early-stage disease [1, 2].

Early-stage ovarian cancer patients have an excellent prognosis with a risk of recurrence of 10–15% at 5 years [3]. Different studies aimed to look for prognostic biomarkers and their integration into clinical practice to identify those women with poor prognosis [4, 5].

Systemic inflammation is linked to cancer initiation, progression, and metastasis [6]; it has been related to cancer mortality [7] and employed as useful prognostic indicator in many solid tumors [8]. Multiple inflammatory markers have been analyzed in patients with gynecological cancers, including the neutrophil–lymphocyte ratio (NLR), platelet-lymphocyte ratio (PLR), eosinophil-lymphocyte ratio (ELR), monocyte-lymphocyte ratio (MLR), systemic immune inflammation index (SII) (defined as platelet x neutrophil–lymphocyte ratio), (eosinophil x neutrophil)/lymphocyte (ENL) and fibrinogen-albumin ratio (FAR) [8–13]. Nevertheless, there is no consensus on which inflammatory marker is mostly related with survival in ovarian cancer. Moreover, only very few studies have included early-stage disease [11, 12] and to our knowledge none of these correlated such biomarkers with BReast CAncer gene (BRCA) mutational status.

The endpoint of the present study was to assess which baseline inflammatory markers have a prognostic impact in early-stage ovarian cancer, and to correlate them with standard prognostic factors and BRCA status.

## Materials and methods

### Inclusion criteria

This is a retrospective, single-center, observational cohort study. The present study was approved by the Institutional Review Board (IRB) of Policlinico Agostino Gemelli IRCCS on 26/05/2020 (number DIPUSVSP-26-05-2076).

All patients with apparent early-stage epithelial ovarian cancer (International Federation of Obstetrics and Gynecology—FIGO I-II and IIIA1) who underwent primary surgery at Policlinico Agostino Gemelli IRCCS between 10/2012 and 12/2019 were included. Patients with diagnosis of another cancer 3 years before ovarian cancer, diagnosis of another cancer after ovarian cancer, non-epithelial ovarian cancer, immunosuppressive drugs, HIV infection or immunosuppressive diseases and those with no information about pre-operative complete blood count (CBC) were excluded. Patients’ data was retrieved from Research Electronic Data Capture (RedCap) institutional database, after IRB approval.

### Inflammatory markers and BRCA status

Inflammatory markers were evaluated on the results of the CBC and coagulation tests, which were performed at the time of the pre-operative anesthetic assessment from 31 days to 1 day before the surgery for ovarian cancer. BRCA status was assessed with germline mutational test after ovarian cancer diagnosis.

### Statistical analysis

Standard descriptive statistics were used to evaluate the distribution of each variable. Continuous variables were reported as median and range, and categorical variables as frequency and percentage. The distribution of variables between groups were compared with chi-square test or Fisher’s exact test, as appropriate. The Receiver Operating Characteristic (ROC) curve was used to determine the optimal cut-off value of different baseline inflammatory biomarkers for the DFS analysis matching the most extreme joint sensitivity and specificity.

DFS was defined as the time interval in months from the date of the ovarian cancer diagnosis to the date of first recurrence or last follow-up. Overall survival (OS) was calculated as the time in months from the date of the diagnosis to the date of the last follow-up or death. DFS and OS were estimated by the Kaplan–Meier method [14] and the log-rank test was used to assess the statistical significance [15]. The impact of different variables on survival, including inflammatory markers, was analyzed using univariate and multivariate Cox proportional hazards models and described using hazard ratios (HRs) and their 95% confidence intervals (95%CI); the Cox regression analysis included the known prognostic factors in ovarian cancer [16, 17]. Multivariate analysis was computed on those factors which resulted significant at univariate analysis.

All *p* values reported are two-sided, and a *p* value < 0.05 was considered statistically significant. Analysis was computed using SPSS version 27.0 (IBM Corporation 2018, Armonk, NY: IBM Corp.).

## Results

### Patients’ characteristics

A total of 359 patients were included in the study period. Patients’ characteristics are showed in Table 1. Most of patients were diagnosed with FIGO-stage IA (*n* = 141, 39.3%), serous histology (*n* = 143, 39.8%) and grade 3 (*n* = 135, 48.7%). Data on BRCA status was available on 127/359 (35.4%) patients. Of these, 40 (31.5%) showed BRCA 1–2 mutation. The median value of pre-operative CA-125 was 45.2 U/mL (5–14,389). Distribution of each inflammatory marker in the present population is reported in Table 1. There was no significant correlation between BRCA status and inflammation markers (Table 2). The only positive correlation NLR ≥ 3 and SII ≥ 1000 was with CA125 ≥ 35 U/mL (*p* < 0.001) (Supplemental Table 1).

**Table 1:** Characteristics of the entire population

	Total
	*N* (%)
All cases	359
Mean age at diagnosis (range, years)	54 (21–93)
CA125, mean (range), UI/mL	69 (5–14,389)
Final FIGO stage	
IA	141 (39.3)
IB	20 (5.6)
IC1	35 (9.7)
IC2	40 (11.1)
IC3	12 (3.3)
IIA	31 (8.6)
IIB	50 (13.9)
IIIA1	30 (8.5)
Histology	
Serous	143 (39.8)
Endometrioid	101 (28.1)
Mucinous	47 (13.1)
Clear cell	57 (15.9)
Undifferentiated	1 (0.3)
Mixed	5 (1.4)
Others	5 (1.4)
Residual tumor at the end of surgery	
No gross residual tumor	
Residual tumor	359 (100.0)
	0
Grading^a^	
1	44 (15.9)
2	98 (35.4)
3	135 (48.7)
Type of BRCA mutation^b^	
No mutation	87 (68.5)
BRCA1 mutation	30 (23.6)
BRCA2 mutation	9 (7.1)
BRCA1-2 mutation	1 (0.8)
Inflammatory markers*	
SII	
< 1000	263 (73.3)
≥ 1000	96 (26.7)
NLR	
< 3	236 (65.7)
≥ 3	123 (34.3)
PLR	
< 200	269 (74.9)
≥ 200	90 (25.1)
ELR	
< 0.03	60 (16.7)
≥ 0.03	299 (83.3)
ENL	
< 0.6	291 (81.1)
≥ 0.6	68 (18.9)
FAR	
< 10	226 (63.0)
≥ 10	133 (37.0)
MLR	
< 0.2	156 (43.4)
≥ 0.2	203 (56.5)

*FIGO* International Federation of Gynecology and Obstetrics; *BRCA* Breast Cancer gene; *NLR* neutrophil-lymphocyte ratio, *PLR* platelet-lymphocyte ratio, *ELR* eosinophil-lymphocyte ratio, *MLR* monocyte-lymphocyte ratio, systemic immune inflammation index (SII = platelet x neutrophil/lymphocyte), *ENL* (eosinophil x neutrophil)/lymphocyte, *FAR* fibrinogen-albumin ratio

*Calculated with ROC curves

^a^Data calculated on 277 patients due to lack of data of 82 patients

^b^Data calculated on 127 patients due to lack of data of 232 patients

**Table 2:** Correlation between BRCA status and systemic inflammatory markers

	BRCA wild type	BRCA mutated	*p* value
SII < 1000	62 (71.3)	26 (65.0)	0.536
≥ 1000	25 (28.7)	14 (35.9)	
NLR < 3	58 (66.7)	21 (52.5)	0.168
≥ 3	29 (33.3)	19 (47.5)	
PLR < 200	61 (70.1)	25 (62.5)	0.419
≥ 200	26 (29.9)	15 (37.5)	
ELR < 0.03	4 (4.6)	4 (10.0)	0.259
≥ 0.03	83 (95.4)	36 (90.0)	
ENL < 0.6	1 (1.1)	2 (5.0)	0.233
≥ 0.6	86 (98.9)	38 (95.0)	
FAR < 10	53 (60.9)	23 (57.5)	0.846
≥ 10	34 (39.1)	17 (42.5)	
MLR < 0.2	12 (13.8)	9 (22.5)	0.303
≥ 0.2	75 (86.2)	31 (77.5)	

On 232 patients BRCA status unavailable

*NLR* neutrophil–lymphocyte ratio, *PLR* platelet-lymphocyte ratio, *ELR* eosinophil-lymphocyte ratio, *MLR* monocyte-lymphocyte ratio, systemic immune inflammation index (SII—platelet x neutrophil–lymphocyte ratio), *ENL* (eosinophil x neutrophil)/lymphocyte, *FAR* fibrinogen-albumin ratio

### Survival analysis

The median follow-up of the entire cohort was 31 months (95%CI 28.5–33.4). The 3 year DFS and OS of the entire population was 82.1% and 97.2%, respectively.

Table 3 demonstrates the univariate Cox regression model comparing the risk of recurrence and death for each inflammatory marker. NLR ≥ 3 and SII ≥ 1000 were associated with significant risk of recurrence and death. PLR ≥ 200 was associated with increased risk of death.

**Table 3:** Cox regression analysis for risk of recurrence and death analyzing the different systemic inflammatory markers

Inflammatory marker	Recurrence		Death	
	HR (95% CI)	*p* value	HR (95% CI)	*p* value
SII < 1000 ≥ 1000	2.150 (1.136–4.071)	**0.019**	4.030 (1.059–15.337)	**0.041**
NLR < 3 ≥ 3	1.917 (1.021–3.599)	**0.043**	5.807 (1.450–23.260)	**0.013**
PLR < 200 ≥ 200	1.466 (0.748–2.876)	0.266	4.279 (1.146–15.981)	**0.031**
ELR < 0.03 ≥ 0.03	2.360 (0.226–2.465)	0.182	0.334 (0.067–1.658)	0.180
ENL < 0.6 ≥ 0.6	0.956 (0.423–2.161)	0.914	0.662 (0.081–5.386)	0.700
FAR < 10 ≥ 10	1.658 (0.893–3.081)	0.109	2.516 (0.535–11.827)	0.243
MLR < 0.2 ≥ 0.2	2.909 (0.897–9.435)	0.075	1.988 (0.247–15.989)	0.518

Bold values are the statistically significant *p*–values

*NLR* neutrophil–lymphocyte ratio, *PLR* platelet-lymphocyte ratio, *ELR* eosinophil-lymphocyte ratio, *MLR* monocyte-lymphocyte ratio, systemic immune inflammation index (SII—platelet x neutrophil–lymphocyte ratio), *ENL* (eosinophil x neutrophil)/lymphocyte, *FAR* fibrinogen-albumin ratio

**Fig. 1:**
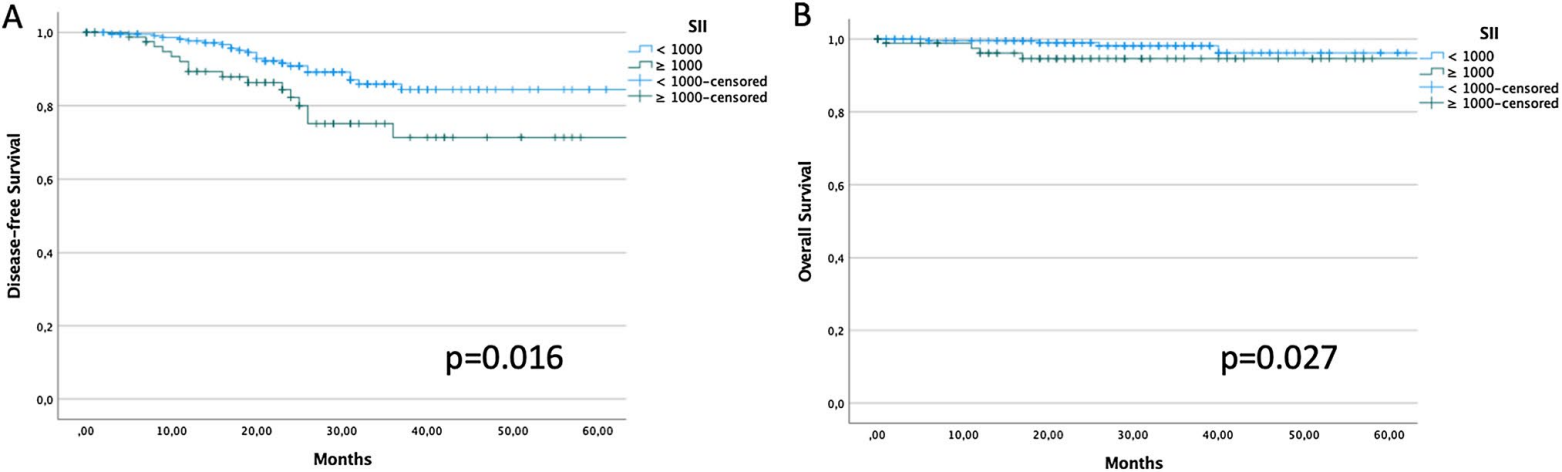
DFS (1A) and OS (1B) stratified according to baseline SII value (cut-off: 1000)

The 3 year DFS of patients with SII < and ≥ 1000 was 85.9% and 71.4%, respectively (*p* = 0.016) (Fig. 1A). The 3 year OS of patients with SII < and ≥ 1000 was 98.2% and 94.6%, respectively (*p* = 0.027) (Fig. 1B). BRCA-mutated patients with SII ≥ 1000 had significantly worse DFS, but not OS, (3 year DFS 31.8 vs 71.1%, *p* = 0.003; 3 year OS 81.8 vs 100%, *p* = 0.179) (Supplemental Fig. 1). SII failed to identify patients with different DFS and OS in the BRCA wild type group (*p* = 0.953 and *p* = 0.807, respectively).

**Fig. 2:**
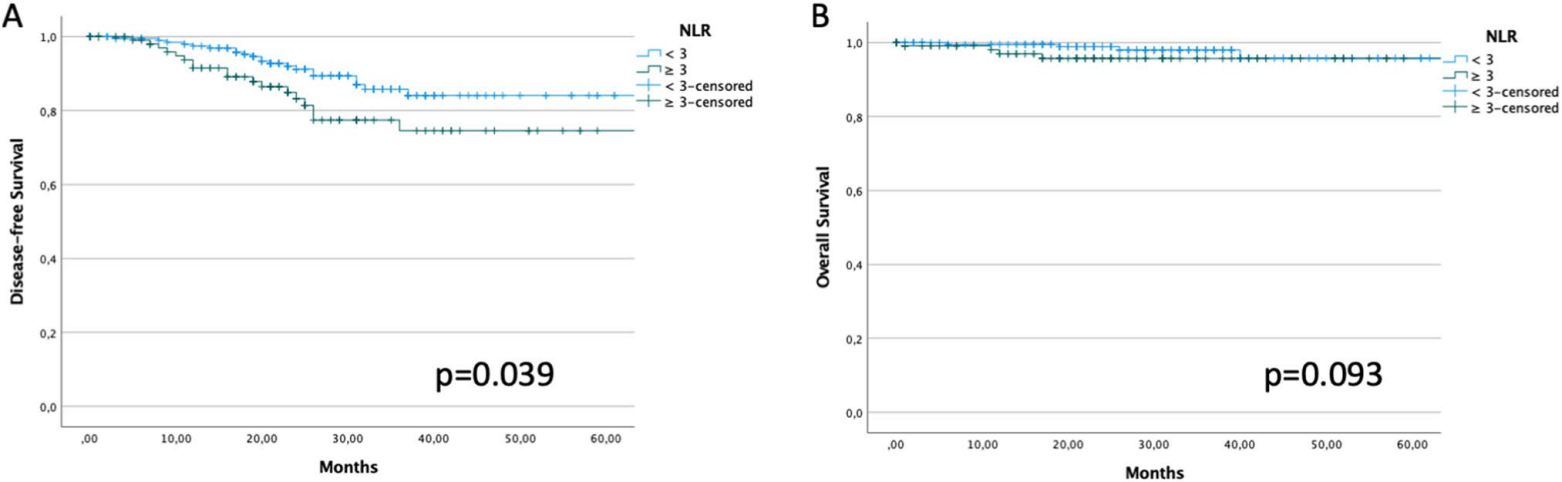
DFS (2A) and OS (2B) stratified according to baseline NLR value (cut-off: 3)

The 3 year DFS of patients with NLR < and ≥ 3 was 85.8% and 74.6%, respectively (*p* = 0.039) (Fig. 2A). The 3 year OS of patients with NLR < and ≥ 3 was 98.0% and 95.7%, respectively (*p* = 0.093) (Fig. 2B). Patients with BRCA mutation and NLR ≥ 3 had significantly worse DFS, but not OS (3 year DFS 48.7 vs 65.6%, *p* = 0.048; 3 year OS 86.7 vs 100%, *p* = 0.515) (Supplemental Fig. 2). NLR ≥ 3 did not show any difference in DFS and OS in BRCA wild type patients (*p* = 0.859 and *p* = 0.368, respectively).

Table 4 demonstrates the univariate and multivariate analyses for risk of recurrence. FIGO stage > I was the only independent risk factor for higher risk of recurrence (HR 2.914, 95%CI 1.323–6.417; *p* = 0.008). No independent variable was identified as predictive of OS in this study population (Supplemental Table 2).

**Table 4:** Univariate and multivariate Cox regression analysis for risk of recurrence analyzing the know prognostic risk factors and systemic inflammatory markers

Characteristic	Univariate		Multivariate	
	HR (95% CI)	*p* value	HR (95% CI)	*p* value
SII < 1000 ≥ 1000	2.150 (1.136–4.071)	**0.019**	2.404 (0.776–5.360)	0.148
NLR < 3 ≥ 3	1.917 (1.021–3.599)	**0.043**	1.179 (0.453–3.066)	0.736
PLR < 200 ≥ 200	1.466 (0.748–2.876)	0.266		
Lymphadenectomy No Yes	0.919 (0.485–1.739)	0.795		
FIGO stage I II/IIIA1	4.077 (2.150–7.731)	** < 0.001**	2.914 (1.323–6.417)	**0.008**
Age at diagnosis < 60 years ≥ 60 years	1.213 (0.630–2.335)	0.563		
Grade 1–2 3	1.615 (1.088–2.396)	**0.017**	1.351 (0.884–2.064)	0.165
Histology Serous Others	0.395 (0.207–0.754)	**0.005**	0.802 (0.357–1.801)	0.592

Bold values are the statistically significant *p*–values

*NLR* neutrophil–lymphocyte ratio, *PLR* platelet-lymphocyte ratio, *ELR* eosinophil-lymphocyte ratio, systemic immune inflammation index (SII—platelet x neutrophil–lymphocyte ratio)

## Discussion

With the present study we showed that high levels of SII and NLR were significantly associated with risk of recurrence and, together with PLR, with risk of death in a population of early-stage ovarian cancer patients. SII ≥ 1000 and NLR ≥ 3 were associated with worse 3 year DFS and baseline SII ≥ 1000 was associated with worse 3 year OS. These results are in line with previous report which reported the prognostic impact of different inflammatory markers in ovarian cancer [9–13]; nevertheless, none of these studies analyzed this specific subset of disease and none compared different markers in the same population.

This result was not confirmed at multivariate analysis, probably due to a potential interaction between these variables, which all include lymphocytes value in their formula.

When investigating why these inflammatory markers had a survival impact instead of others, we found contrasting results with different studies showing the pro-tumorigenic effect of neutrophils and platelets [18, 19], while others reporting that lymphocytes, facilitate antitumor immunity [20, 21]. Emerging evidence indicates the involvement of neutrophils in cancer initiation, progression and metastasis and that platelets enhance tumor cell dissemination by activating endothelial cell function and recruiting immune cells to primary and metastatic tumor sites [19, 22]. Our results support these theories according to which neutrophils and platelets would have a tumorigenic and lymphocytes an antitumor effect.

It is known that BRCA-mutated ovarian cancer exhibits significantly higher mutational and neoantigen loads with higher inflammatory burden than BRCA wild type [23]. In a previous series of advanced ovarian cancer, we showed that patients with lower levels of baseline NLR had better DFS in both BRCA mutated and wild type groups (with borderline significance in the wild type group) [23]. Results on DFS in BRCA mutated subgroups is in line with our results. On the other hand, we did not find any survival difference in BRCA wild type subgroups according to different levels of baseline inflammation markers. However, survival results according to BRCA mutation in the present series must be interpreted with caution. The information about BRCA status was known in only 35.4% of the entire population and the number of events in these subgroups of patients might be statistically underpowered to draw solid conclusions. Further studies focusing on the relation between systemic inflammation and BRCA mutational status may be needed to potentially identify a subgroup of patients with worse survival.

Multivariate analysis demonstrated FIGO stage > I as independent variable associated with risk of recurrence and did not identify any independent variable associated with risk of death; again, this may be related to a potential interaction between the different inflammatory markers but also to the low number of deaths in the entire cohort.

The value of the present study is represented by the identification of a subset of patients at higher risk of recurrence and death; this group of women with apparent early-stage ovarian cancer may be the target for additional/targeted therapies and a closer follow up.

Additionally, inflammatory markers have been reported to be not only a prognostic marker, but also a diagnostic aid to discriminate the risk of malignancy of an ovarian mass. A recent study reported a promising accuracy of inflammatory markers to define the risk of malignancy [12]. In this context, it would be interesting to further analyze whether these markers may be diagnostic of recurrence and if a simple CBC may help to identify recurrent disease.

Limitations of the present study are represented by its retrospective design, leading to inherit potential selection bias as well as the number of missing BRCA tests. Moreover, cut-off values for each inflammatory marker were designed on the ROC curve for DFS with a potential bias when analyzing the same cut-off for OS. However, we did not consider OS in our endpoints in view of the low number of deaths in the present series, with relatively good prognosis. On the other hand, to best of our knowledge, this is the first study comparing the prognostic impact of different inflammatory markers in newly diagnosed early-stage ovarian cancer. Moreover, for the first time we report the prognosis of combination of inflammatory markers and BRCA mutational status in this setting.

## Conclusion

In a population of patients with apparent early-stage ovarian cancer, baseline SII ≥ 1000 and NLR ≥ 3 were associated with worse 3 year DFS and baseline SII ≥ 1000 was associated with worse 3 year OS. The subgroups of BRCA-mutated patients and baseline elevated inflammation markers (SII ≥ 1000 and NLR ≥ 3) were associated with the worse DFS. The only independent factor associated with increased risk of recurrence was FIGO stage > I. The stratification of patients according to NLR and SII at diagnosis may be helpful in defining the need for personalized treatment and more intensive surveillance.

## Supplementary Information

Below is the link to the electronic supplementary material.

Supplementary file1 (DOCX 12634 KB)

## Data Availability

The datasets generated during and/or analysed during the current study are available from the corresponding author on reasonable request.
