# Supplementary material for: The prognostic role of systemic inflammatory markers in apparent early-stage ovarian cancer
Source: Int J Clin Oncol. 2022 Nov 22;28(2):314–20. doi: 10.1007/s10147-022-02272-z (PMC9889507; doi:10.1007/s10147-022-02272-z)

**Supplemental Table 1.** Correlation between clinic-pathologic characteristics and systemic inflammatory markers

|  | SII < 1000 | SII ≥ 1000 | p-value |
| --- | --- | --- | --- |
| Age  < 60 years  ≥ 60 years | 164 (62.4)  99 (37.6) | 66 (68.8)  30 (31.3) | 0.320 |
| CA125  < 35 U/mL  ≥ 35 U/mL | 91 (43.3)  119 (56.7) | 13 (15.7)  70 (84.3) | **<0.001** |
| FIGO stage  I  II/IIIA1 | 189 (71.9)  74 (28.1) | 59 (61.5)  37 (38.5) | 0.071 |
| Lymph node metastasis  No  Yes | 243 (92.4)  20 (7.6) | 86 (89.6)  10 (10.4) | 0.394 |
| Grade  1-2  3 | 103 (42.2)  141 (57.8) | 39 (45.3)  47 (54.7) | 0.615 |
| Histology  Serous  Non-serous | 111 (42.2)  152 (57.8) | 33 (34.4)  63 (65.6) | 0.224 |
|  | NLR < 3 | NLR ≥ 3 | p-value |
| Age  < 60 years  ≥ 60 years | 147 (62.3)  89 (37.7) | 83 (67.5)  40 (32.5) | 0.355 |
| CA125  < 35 U/mL  ≥ 35 U/mL | 81 (42.9)  108 (57.1) | 23 (22.1)  81 (77.9) | **<0.001** |
| FIGO stage  I  II/IIIA1 | 171 (72.5)  65 (27.5) | 77 (62.6)  46 (37.4) | 0.071 |
| Lymph node metastasis  No  Yes | 215 (91.1)  21 (8.9) | 114 (92.7)  9 (7.3) | 0.691 |
| Grade  1-2  3 | 97 (44.1)  123 (55.9) | 45 (40.9)  65 (59.1) | 0.638 |
| Histology  Serous  Non-serous | 100 (42.4)  136 (57.6) | 44 (35.8)  79 (64.2) | 0.257 |

**Supplemental Table 2.** Univariate and multivariate Cox regression analysis for risk of death analyzing the know prognostic risk factors and systemic inflammatory markers.

|  | UNIVARIATE | | MULTIVARIATE | |
| --- | --- | --- | --- | --- |
| Characteristic | HR (95% CI) | p-value | HR (95% CI) | p-value |
| SII  < 1000  ≥ 1000 | 4.030 (1.059-15.337) | **0.041** | 2.377 (0.214-26.400) | 0.481 |
| NLR  < 3  ≥ 3 | 5.807 (1.450-23.260) | **0.013** | 0.929 (0.096-9.018) | 0.949 |
| PLR  < 200  ≥ 200 | 4.279 (1.146-15.981) | **0.031** | 2.699 (0.515-14.132) | 0.240 |
| Lymphadenectomy  No  Yes | 0.365 (0.076-1.762) | 0.210 |  |  |
| FIGO stage  I  II/IIIA1 | 1.355 (0.333-5.511) | 0.671 |  |  |
| Age at diagnosis  < 60 years  ≥ 60 years | 1.041 (0.260-4.165) | 0.955 |  |  |
| Grade  1-2  3 | 0.906 (0.452-1.816) | 0.781 |  |  |
| Histology  Serous  Others | 1.460 (0.360-5.911) | 0.596 |  |  |
| BRCA status  BRCAwt  BRCAmut | 2.040 (0.408-10.210) | 0.386 |  |  |

**Supplemental Figure 1.** DFS and OS stratified according to baseline SII value (cut-off: 1000) and BRCA mutational status.


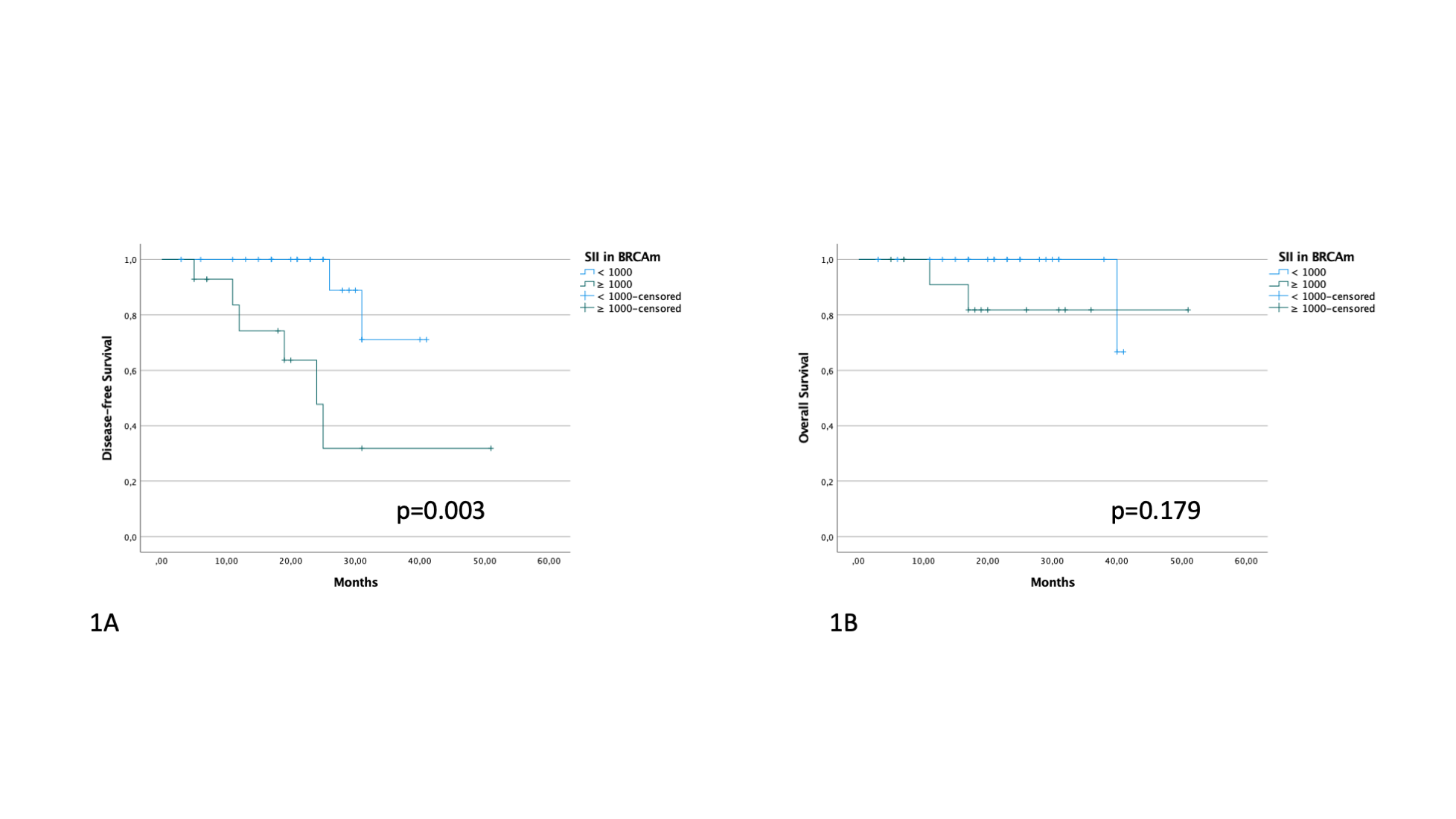


**Supplemental Figure 2.** DFS and OS stratified according to baseline NLR value (cut-off: 3) and BRCA mutational status


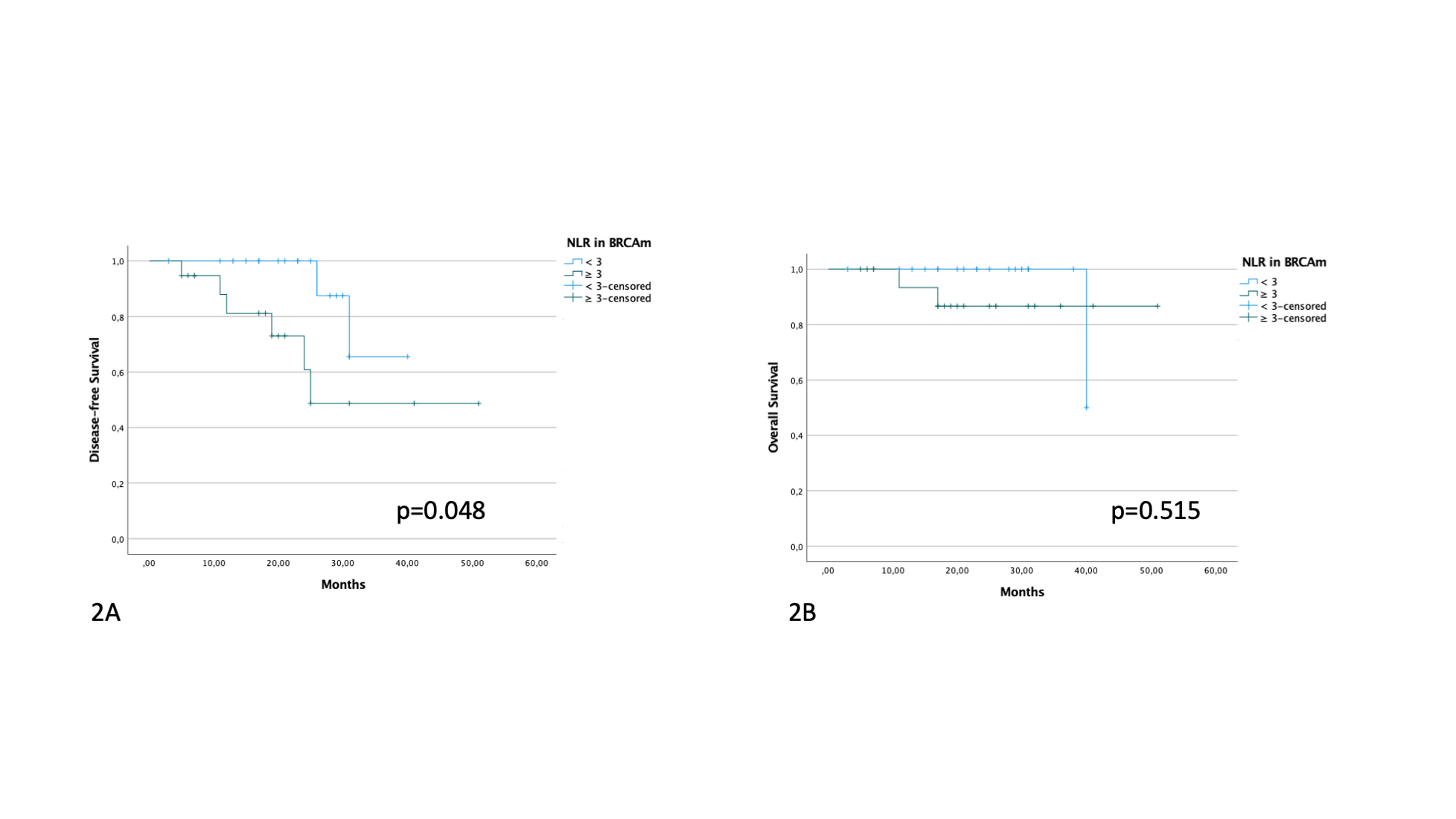

Supplement: Supplementary file 1 — Supplementary file1 (DOCX 12634 KB) [file 10147_2022_2272_MOESM1_ESM.docx]
